# Wild-Type Isocitrate Dehydrogenase-Dependent Oxidative Decarboxylation and Reductive Carboxylation in Cancer and Their Clinical Significance

**DOI:** 10.3390/cancers14235779

**Published:** 2022-11-24

**Authors:** Qiwei He, Junxiong Chen, Zijing Xie, Zhenzhou Chen

**Affiliations:** Neurosurgery Center, Department of Neuro-Oncological Surgery, The National Key Clinical Specialty, The Engineering Technology Research Center of Education Ministry of China on Diagnosis and Treatment of Cerebrovascular Disease, Guangdong Provincial Key Laboratory on Brain Function Repair and Regeneration, The Neurosurgery Institute of Guangdong Province, Zhujiang Hospital, Southern Medical University, Guangzhou 510282, China

**Keywords:** isocitrate dehydrogenase (IDH), cancer metabolism, oxidative decarboxylation, reductive carboxylation, α-ketoglutarate (α-KG), nicotinamide adenine dinucleotide phosphate (NADPH)

## Abstract

**Simple Summary:**

While isocitrate dehydrogenases have been known for decades, their mechanisms in tumors are gradually becoming clearer. The three isocitrate dehydrogenase isoforms in human cells differ in their subcellular localization, molecular structure, cofactor requirement, and catalytic mechanism. Here, we review the role of isocitrate dehydrogenases in metabolism, provide a current overview of cancer-linked isocitrate dehydrogenases and point to future lines of research to better understand the complex biology in health and disease.

**Abstract:**

The human isocitrate dehydrogenase (IDH) gene encodes for the isoenzymes IDH1, 2, and 3, which catalyze the conversion of isocitrate and α-ketoglutarate (α-KG) and are required for normal mammalian metabolism. Isocitrate dehydrogenase 1 and 2 catalyze the reversible conversion of isocitrate to α-KG. Isocitrate dehydrogenase 3 is the key enzyme that mediates the production of α-KG from isocitrate in the tricarboxylic acid (TCA) cycle. In the TCA cycle, the decarboxylation reaction catalyzed by isocitrate dehydrogenase mediates the conversion of isocitrate to α-KG accompanied by dehydrogenation, a process commonly known as oxidative decarboxylation. The formation of 6-C isocitrate from α-KG and CO_2_ catalyzed by IDH is termed reductive carboxylation. This IDH-mediated reversible reaction is of great importance in tumor cells. We outline the role of the various isocitrate dehydrogenase isoforms in cancer, discuss the metabolic implications of interference with IDH, summarize therapeutic interventions targeting changes in IDH expression, and highlight areas for future research.

## 1. Introduction

Research on isocitrate dehydrogenase (IDH) in cancer is divided into *wild-type IDH* and *mutant IDH* [1]. Many previous studies have treated tumors as a single entity without distinguishing between mutant and wild-type IDH. However, it is now widely accepted that *wild-type IDH* and *mutant IDH* tumors are fundamentally different [1]. In recent years, research on IDH in tumors has focused on the mutant types, and many effective inhibitors of mutant-IDH have been used clinically with promising results [1]. Mutant IDH decreases α-KG production, reduces NADPH from isocitrate and NADP^+^, and catalyzes the conversion of α-KG to D-2-hydroxyglutarate (D-2HG) [2]. Elevated D-2HG is proposed to promote tumorigenesis [1]. IDH1 and IDH2 mutations result in active site substitutions that profoundly affect IDH activity, cellular metabolism, and cancer development [1,3,4]. Meanwhile, the study of wild-type-IDH in tumors has made significant progress in recent years. Several studies have shown that increased expression of *wild-type-IDH* in tumors has a worse prognosis than increased *mutant-IDH* [5]. 90% of gliomas have *wild-type IDH*, with a worse prognosis than *mutant IDH* [5]. Wild-type IDH enzymes play a key role in promoting tumor proliferation and recurrence in therapy. Therefore, it is crucial to elucidate the mechanism of wild-type IDH in tumors.

Under normal conditions, cells require an adequate oxygen concentration (O_2_) for aerobic respiration, producing adenosine triphosphate (ATP), the most direct energy source for living organisms. Although highly proliferating tumor cells consume much glucose, their production of ATP is inefficient [6,7,8,9]. This is because rapidly proliferating cells do not need to produce much ATP to carry out their cellular functions but must synthesize biomolecules such as nucleotides, amino acids, and fatty acids to promote cell division and growth [6,7,8]. The reductive carboxylation provides a shortcut for this process of anabolism [10,11]. IDH1 and IDH2 mediate the direct generation of isocitrate from α-KG in the reductive carboxylation reaction [3], and some scholars describe this reaction process as reductive TCA or “counter-clockwise” TCA [12,13,14]. Interestingly, some studies have advocated that the reductive carboxylation catalyzed by IDH1 and 2 is inconsequential as it converts the α-KG back to isocitrate, creating an “ineffective cycle” [9].

Many studies have shown that it is a vital metabolic pathway, especially in rapidly proliferating tumor cells. For example, it was found that the growth rate of tumor cells is reduced when they are deficient in glutamine or when they are grown in a medium without α-KG [15,16]. Increased glutamine consumption by tumor cells leads to an increase in glutamine-derived isocitrate, which in turn produces more citrate [10,11]. Citrate cannot cross the inner mitochondrial membrane freely and must enter the cytoplasm via the citrate malate pyruvate cycle [17]. ATP citrate lyase catalyzes the reversible conversion of citrate to acetyl-CoA, the raw material for fatty acid synthesis [17]. Fatty acids are key to the formation of new membranes and essential for the rapid proliferation of cells [10]. IDH-mediated isocitrate oxidative decarboxylation is also of great significance. On the one hand, it is an important part of the TCA cycle and provides hydrogen atoms for the respiratory chain to generate ATP. On the other hand, the NAD(P)^+^/NAD(P)H redox ratio transition is altered to adapt to changes in cellular metabolism to perform other functions, which are discussed below [18]. Therefore, we review the current knowledge of wild-type IDH enzymes and their potential role in the mechanisms driving tumor progression. We aim to provide insights for studying reprogramming the metabolism of tumor cells to inhibit their proliferation and survival.

## 2. Catalytic Mechanism and Enzyme Structure of IDHs

**Figure 1:**
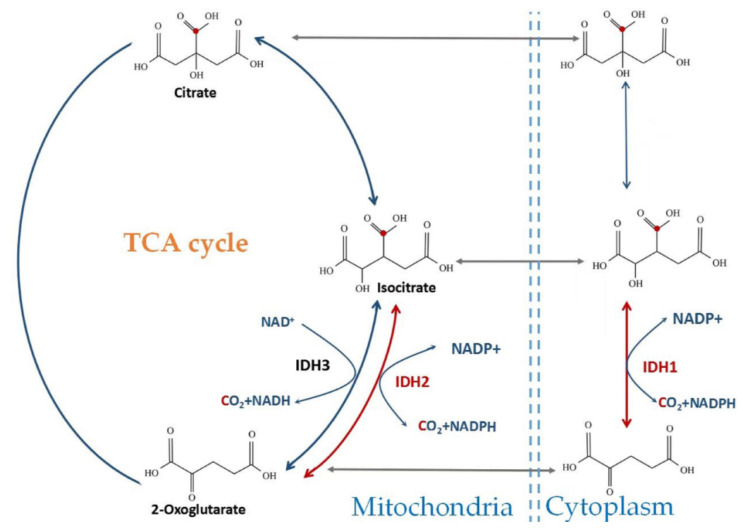
Subcellular localization and chemical reactions catalyzed by wild-type IDH. IDH1 is localized in the cytosol; IDH2 and IDH3 are localized in the mitochondrial matrix. IDH1/2 reversibly oxidizes isocitrate to α-KG and CO_2_ with the formation of NADPH. IDH3 irreversibly oxidizes isocitrate to α-KG with the formation of NADH as part of the TCA cycle.

The expression of the three IDH isoforms in human cells differs in their subcellular localization, molecular structure, cofactor requirement, and catalytic mechanism (Figure 1) [19]. IDH1 is localized in the cytoplasm and peroxisomes, whereas IDH2 and IDH3 are found in the mitochondria [20]. *IDH1* and *IDH2* (EC code: 1.1.1.42) share considerable sequence similarity (70% identity in humans) and catalyze the same reaction [20,21]. Reductive carboxylation of αKG to isocitrate by NADPH and CO_2_ can be catalyzed by IDH1 and IDH2, which catalyze reversible reactions [19,20,21]. Reversible CO_2_ fixation was also observed by isotopic analysis of TCA cycle intermediates in rat liver [22]. 

IDH1 exists as a homodimer with a clamp structural domain, two large structural domains, and two small structural domains per IDH1 monomer. The large and small structural domains are linked by a β-sheet [23]. IDH2 and IDH1 are isozymes with very similar structures [20]. In the TCA cycle, IDH3 (EC code: 1.1.1.41) uses NAD^+^ as a cofactor to catalyze the reaction to form α-KG and NADH. The reaction is irreversible, and IDH3 is allosterically regulated by citrate, adenosine diphosphate, and adenosine triphosphate [24]. α-KG is further metabolized to succinate, whereas NADH is oxidized by the electron transport chain [25]. IDH3 heterodimers are composed of αβ- and αγ-subunits, which assemble to form α2βγ heterotetramers [24]. The IDH3α-subunit is encoded by the *IDH3A* gene (chromosome 15q25.1), the β-subunit by the *IDH3B* gene (chromosome 20p13), and the γ-subunit by the *IDH3G* gene (chromosome Xq28) [24]. The α subunit is the catalytic subunit [26], and the β and γ subunits are the regulatory subunits [27]. αβ and αγ alone have basal activity, but the full activity of IDH3 needs the assembly and synergistic function of these two heterodimers [24].

## 3. The Role of Wild-Type IDH1 and IDH2 in Cellular Metabolism

**Figure 2:**
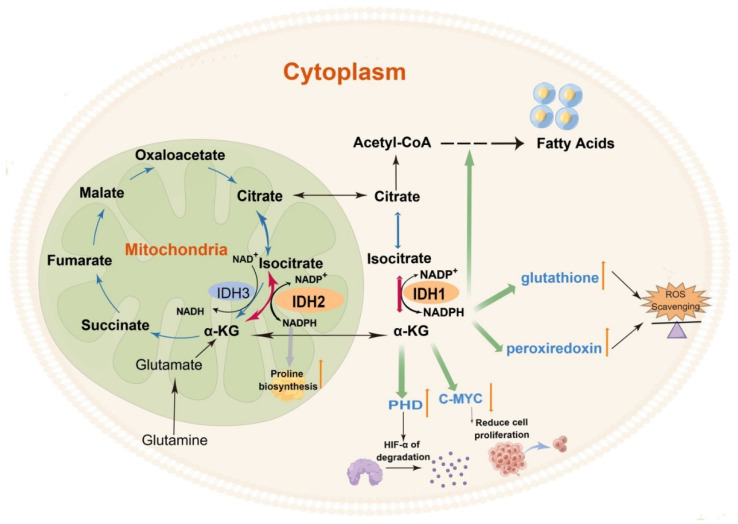
The role of wild-type IDH in human cells. The reaction catalyzed by IDH1 and IDH2 provides NADPH to maintain the pool of reduced glutathione and peroxiredoxin that counteract reactive oxygen species (ROS). The reaction catalyzed by IDH1 and IDH2 provides α-KG to increase the activity of the prolyl hydroxylase domain (PHD) and downregulate c-Myc expression. IDH3, the key enzyme of the TCA cycle, serves as the central regulatory site of the TCA cycle. (By Figdraw).

An increasing number of studies have shown that although IDH1 and 2 appear to catalyze the same reaction as IDH3 to form αKG from isocitrate, they have different effects. The reversible reactions catalyzed by IDH1 and IDH2 are accompanied by the production and consumption of the reducing agent NADPH and the binding and release of CO_2_, respectively, which in turn has multiple effects on the cell (Figure 2).

Oxidative TCA catalyzed by IDH1 and IDH2 is accompanied by the production of NADPH to balance the cellular redox equilibrium [28] and maintain the pool of reduced glutathione and peroxiredoxin that counteract reactive oxygen species (ROS) [29], protecting cells from oxidative damage, lipid peroxidation, and DNA damage [29,30]. Craig B. Thompson’s team found that the primary physiological function of NADPH in mitochondria is to promote the biosynthesis of the non-essential amino acid proline [12]. They also showed that mitochondria are the sites of proline biosynthesis. Pyrroline-5-carboxylate synthase (P5CS) catalyzes the conversion of glutamine-derived glutamate to pyrroline-5-carboxylate (P5C) [12]. P5C is further reduced to proline by mitochondrial pyrroline-5-carboxylate reductases (PYCR1 and PYCR2). P5CS is an NADPH-dependent metabolic enzyme; consequently, decreased NADPH levels inhibit proline biosynthesis derived from glutamine [12]. As NADP^+^ and NADPH cannot cross the intracellular membrane, cytoplasmic and mitochondrial NADP(H) cannot circulate freely [31,32,33]. IDH1 in the cytoplasm can produce non-mitochondrial NADPH, which also acts as an antioxidant to protect cells from oxidative stress [34]. IDH1-deficient mice exhibit increased accumulation of ROS in the liver, leading to DNA damage and subsequent induction of apoptosis [35]. In addition, NADPH produced in the cytoplasm also acts as a key-reducing equivalent for lipid synthesis. The reverse/reductive TCA is critical for glucose- and glutamine-stimulated insulin secretion. The mechanism mainly involves that IDH2 can catalyze the reverse TCA reaction leading to increased isocitrate and citrate, which then drives their oxidation by IDH1 in the cytosol to produce NADPH. The cytosolic NADPH activates sentrin/SUMO-specific protease-1 (SENP1), which promotes insulin granule exocytosis [14]. The reductive TCA catalyzed by IDH2 is found mainly in humans’ retinal pigment epithelium (RPE), which utilizes the cellular NADPH to protect itself from oxidative stress [18]. Support of reductive carboxylation by supplementation with NAD^+^ precursor or the substrate α-KG or treatment with poly (ADP ribose) polymerase (PARP) inhibitors could protect RPE from excessive oxidative stress, providing an effective therapeutic strategy for the treatment of blinding diseases caused by RPE dysfunction [18]. They further demonstrated that both NAD^+^ precursor and PARP inhibitors increase NAD^+^ levels and, in turn, NADH. NADH is then converted to NADPH, which acts as a coenzyme for IDH2 to catalyze the reductive carboxylation reaction [18]. The pentose phosphate pathway (PPP) also produces NADPH, which is repurposed to counteract cytosolic ROS in the absence of IDH1 [16]. Besides, cytosolic and mitochondrial folate metabolism both contribute to NADPH homeostasis. Consistent with folate metabolism being a significant NADPH producer, antifolates have been shown to induce oxidative stress [36].

Carbon (C) is the most basic element that makes up the macromolecules of all organisms. Forward TCA is a catabolic pathway, while IDH1- and IDH2-mediated reverse TCA is the anabolic process of adding H and C. This process, known as reductive carboxylation, involves adding C of CO_2_ directly to α-KG to form 6-C isocitrate, which isomerizes to form citrate. Studies have confirmed that the active IDH2-mediated reductive TCA cycle in acute myeloid leukemia (AML) cells promotes the conversion of α-KG to isocitrate/citrate, thereby facilitating the synthesis of lipids from glutamine [13]. 

## 4. The Role of Wild-Type IDH3 in Cellular Metabolism

IDH3, the key enzyme of the TCA cycle, together with citrate synthase and α-ketoglutarate dehydrogenase, serves as the central regulatory site of the TCA cycle, mainly by feedback inhibition of the product. The TCA cycle is the cells’ most critical process for energy production, so the ratio of ATP/ADP to NAD^+^/NADH is also important to regulate the enzymes that ultimately sense cellular ATP and NADH concentration to ensure cellular energy supply. NAD^+^/NADH balance is fundamental to metabolism [37,38,39]. Glycolysis, fatty acid oxidation, amino acid degradation, and citric acid cycle require NAD^+^ as a coenzyme [37,38,39,40]. The generated NADH transfers electrons to oxygen through the electron transport chain, producing ATP and NAD^+^, a process called NAD^+^ recycling. When mitochondrial respiration is impaired, the cytosolic pyruvate reduces to lactate and produces NAD^+^. Moreover, when the cytoplasmic NAD^+^/NADH ratio decreases, pyruvate reduces to lactate, converting NADH to NAD^+^, which is the most crucial replenishing pathway for the cytoplasmic pool of NAD^+^. The delta-5 and delta-6 desaturases (D5D/D6D) (key enzymes responsible for highly unsaturated fatty acid (HUFA) synthesis) can convert NADH into NAD^+^ to maintain cell proliferation, which is also an NAD^+^ cycling mechanism similar to the glycolytic flux. Inhibition of HUFA synthesis or lactic acid fermentation increases the other to compensate for the absence of the NAD^+^ cycle, highlighting their interdependence [41]. NAD^+^ is also involved in DNA repair as a substrate for PARP; PARP-1, PARP-2, and PARP-3 of the PARP family are involved in DNA repair [42]. Thus, IDH3 is not only a key enzyme of the TCA cycle but also plays a role in regulating the ATP and NADH levels, which in turn regulates cell functions to adapt to environmental changes.

## 5. Research Progress of Wild-Type IDH1 in Cancers

Molecular genetic markers of tumors are closely related to patient outcome and prognosis, and there is increasing evidence that IDHs play a key role in cancer metabolism. In recent years, *mutant IDHs* have been increasingly studied.

Mutant phenotypes are common in numerous tumors, such as chondrosarcomas, hepatocellular carcinomas, acute myeloid leukemia, intrahepatic cholangiocarcinomas, and thyroid cancer [43,44,45,46]. Moreover, the vast majority of grade II/III gliomas and secondary glioblastomas (grade IV) are linked to better survival with mutations in IDH1 and IDH2 than wild type [47]. Mutant IDHs differ from wild-type IDH in that mutant IDH converts α-KG to d-2-hydroxyglutarate (D-2-HG), impairs α-KG levels, and consumes NADPH. The expression level of D-2-HG is markedly elevated in malignant tumors, and elevated D-2HG levels promote tumorigenesis [48,49,50,51]. The structure of D-2HG is similar to α-KG and can competitively inhibit α-KG-dependent dioxygenase [52], which is essential for regulating the metabolic and epigenetic state of cells. Inhibitors of mutant IDHs can reduce D-2HG-induced DNA and histone hypermethylation, thereby reducing tumor growth. However, the mechanism of mutant IDH in tumorigenesis is still unclear. Although mutant IDH catalyzes α-KG to D-2-HG, other intermediates in the TCA cycle remain relatively unchanged except for α-KG [53]. α-KG can be replenished by other pathways such as glutamine catabolism [54]. The discovery of elevated D-2-HG in gliomas has opened up new avenues to study the unique metabolites in tumor cells [54]. The effects of the mutant IDHs on cells are limited to elevated levels of specific metabolites and stable levels of other metabolites. In contrast, elevated or reduced wild-type IDHs have significant effects on overall cellular metabolism, which may partly explain the lower malignancy of the mutant IDHs compared to the wild-type IDHs; however, further studies are required to understand the mechanism better (Table 1). 

**Figure 3:**
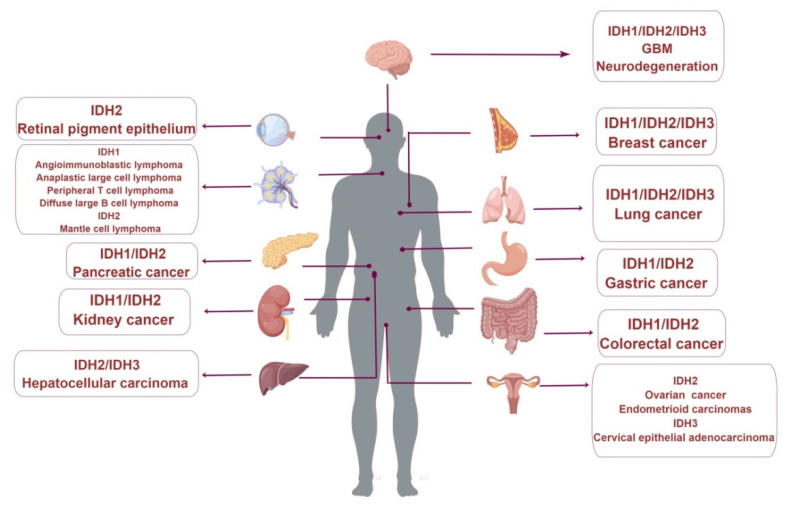
Changes in wild-type IDH expression are associated with human diseases. (By Figdraw).

The effect of wild-type IDH on tumors has become a research focus in recent years (Figure 3). Studies in lung adenocarcinoma patients show increased expression levels of wild-type IDH1, CD44, and reduced glutathione (Figure 3). CD44 is a marker of cancer stem cells involved in tumorigenesis, progression, and metastasis [55]. Glutathione is an important antioxidant and a free radical scavenger in the body that converts harmful intracellular toxins into harmless substances. IDH1 positively correlates with CD44 and reduced glutathione in lung adenocarcinomas, suggesting a positive effect of IDH1 on tumor growth [55]. IDH1 expression was upregulated in several hematologic malignancies, including angioimmunoblastoma, mesenchymal large-cell lymphoma, peripheral T-cell lymphoma, and diffuse large B-cell lymphoma (DLBCL) [57]. Elimination of IDH1 in human acute promyelocytic leukemia (APL) resulted in a phenotype similar to that observed with griseofulvin B (GCN B) treatment, one of the most common active secondary metabolites with potent antitumor activities. This indicates that IDH1 is one of the antitumor targets of GCN B and suggests that wild-type IDH1 may be a potential target for future intervention in hematologic malignancies [69].

Inhibition of IDH1 in leukemia using wild-type IDH1 inhibitors causes a shift in cellular metabolism from oxidative phosphorylation to glycolysis, promoting the Warburg effect, a well-known feature of rapidly proliferating cells in mammals [70]. Tumors in vivo are comprised of at least two kinds of metabolic zones, hypoxic and normoxic zones, defined by the differential uptake of glucose and differential uptake of oxygen [71]. Due to the differential solubility of oxygen and glucose in the blood, cells far from well-perfused capillaries become hypoxic but can still receive glucose [72,73]. The expression of IDH1 in renal cell carcinoma is significantly lower than that in adjacent tissues, and the decreased expression of IDH1 may affect the development of renal cell carcinoma through hypoxia signaling in vitro and in vivo; the overexpression of IDH1 can reduce the expression of HIF-1α and HIF-2α proteins, subsequently inhibiting cell proliferation [56].

The function of IDH1 in tumors is related to the production of the coenzyme NADPH. The expression of wild-type IDH1 is upregulated in 65% of primary glioblastomas (GBM). Silencing of IDH1 slows GBM growth, prolongs the survival of mice carrying patient-derived xenografts, and promotes tumor cell differentiation and tumor cell apoptosis [58]. Inhibition of IDH1 lowers αKG and NADPH levels, thereby decreasing lipid biosynthesis and promoting histone methylation and expression of differentiation marker proteins [58]. In the absence of glucose in mouse mammary tumors, malic enzyme 1/IDH1 knockdown in combination strongly inhibited tumor growth by reducing NADPH production [74]. Decreased IDH1 expression leads to a decrease in NADPH in cells, which promotes consequent exhaustion of glutathione (GSH), as well as the generation of ROS and loss of mitochondrial membrane potential, leading to apoptosis in leukemic cells. IDH1 also regulates one-carbon metabolism in leukemic cells. Knockdown of IDH1 increases metabolites involved in one-carbon metabolisms, such as methionine, taurine, SAM, methylcytosine, and ornithine levels [70].

Pancreatic ductal adenocarcinoma (PDAC) cells can survive under severe conditions, such as low nutrient conditions and chemotherapy, which can be attributable to the rapid mobilization of an antioxidant defense program. The NADPH-generating enzyme IDH1 is a critical regulatory target under the control of HuR [59]. HuR, an RNA-binding protein, is expressed widely in all proliferating cells upon acute stress and has been reported to perform regulatory functions under oxidative stress [75,76]. IDH1 was also shown to be a direct target gene of *hepatocyte nuclear factor 4α (HNF4α)* [77], which is specifically overexpressed in gastric cells (GC) and is functionally required for GC development. Colorectal cancer (CRC) is one of the most common malignancies and a worldwide cause of morbidity and mortality [78]. Positive expression of IDH1 significantly correlates with lymph node metastasis and TNM staging in CRC. Patients with high IDH1 levels are more likely to be invasive and metastatic, for reasons that need further investigation, possibly due to alterations in NADPH levels [79].

## 6. Research Progress of Wild-Type IDH2 in Cancers

Some hypoxic cells can maintain cell proliferation despite a substantial decrease in glucose-derived citrate, with glutamine becoming the primary source of citrate. Glutamine-derived α-KG is reduced and carboxylated by NADPH-dependent mitochondrial IDH2 to form isocitrate, which is then isomerized to citrate. In profound hypoxia (0.5% O_2_), IDH2 carboxylates α-KG to citrate to support glioblastoma cell growth and viability [15,16]. The accumulation of citrate or exogenous citrate in the cytoplasm significantly enhances the migration and invasion of triple-negative breast cancer (TNBC) cells under hypoxia (1% O_2_). The mechanism mainly involves the activation of the AKT/ERK/MMP2/9 signaling axis stimulated by citrate [64].

It is well known that the expression of genes encoding enzymes, in turn, regulates metabolic pathways. Hypoxia-inducible factor (HIF-1) promotes ubiquitination of α-ketoglutarate dehydrogenase (α-KGDH) and protein hydrolysis, resulting in decreased α-KGDH activity and increased α-KG levels, thereby driving the reverse reaction of IDH and promoting lipids production for cell growth and proliferation. HIF-1 can also increase glutamine uptake, glutamate to α-KG flux, and ATP production by upregulating glutamate dehydrogenase (GDH) expression in lung cancer cells. HIF1α binds to the promoter of GDH and promotes GDH gene transcription in lung cancer cells [15,60,80]. However, in recent years, metabolic reprogramming is becoming increasingly popular, a “negative feedback loop” that allows rapid adaptation to the organisms’ needs without changing the gene sequence and, thus, better survival in different environments [15,60,80]. IDH2-mediated conversion of isocitrate and α-KG results in metabolic intermediates that regulate gene expression and lead to a better adaptation to environmental demands. Inhibition of IDH2 with AGI-6780 in mice carrying AML xenografts demonstrated significant therapeutic effects and potential clinical applications. The study also suggested that IDH2 inhibition could transform cancer treatment from cytotoxic to precision therapy and fundamentally change the cancer treatment landscape [13]. Recent research has found that shRNA inhibition of wild-type IDH2 increased α-KG, decreased *c-Myc* (an important oncogene), and suppressed AML viability and proliferation [13]. Prolyl hydroxylase domain enzymes (PHDs) rely on oxygen (O_2_), iron (Fe^2+^), α-KG, and ascorbic acid (vitamin C), to hydroxylate HIF-1α, promoting the degradation of HIF-1α by proteases. HIF-1α is a transcription factor whose expression is increased in various cancers and regulates apoptosis, cell survival, and angiogenesis [81]. The α-KG-dependent oxygenase performs demethylation in cells, which promotes mRNA stability and stable translation and regulates the pluripotency and differentiation potential of embryonic stem cells [82,83]. Wild-type IDH2 is upregulated in lung cancer. Downregulating IDH2 leads to increased α-KG and consequently decreased HIF-1α expression, reducing lung cancer cell proliferation and promoting cellular glucose uptake and lactate production [61].

Various cancers differ in the expression of IDH2, which may be related to the cancer type and staging [62,66]. IDH2 levels are also upregulated in ovarian cancer and endometrioid carcinoma of the endometrium [62,66]. Elevated IDH2 levels are a possible marker of ovarian endometrioid carcinomas. Arsenic trioxide (As2O3), a promising chemotherapeutic agent for cancer treatment, activates long-stranded non-coding RNA in bladder cancer cells. OTUD6B-AS1 lncRNA induces oxidative stress to increase cancer cell damage, specifically by increasing metal-regulated transcription factor 1 (MTF1), which inhibits the expression of mitochondrial NADP^+^-dependent IDH2 [84]. Correlation studies showed that breast and pancreatic cancer patients with high IDH2 levels exhibit worse Overall Survival (OS) and Progression-Free Survival (PFS) [61]. Acetylation of proteins is now recognized as a major post-translational modification for regulating their function. Proteomic analysis indicated that IDH2 is one of many proteins in the mitochondria with acetylation at lysine residues [85], suggesting that the function of IDH2 might be regulated by acetylation. Recent evidence supports that NAD^+^-dependent protein deacetylase, SIRT3, increases IDH2 activity via deacetylation in cancer cells [86]. Lower SIRT3 protein expression was associated with poorer OS in mantle cell lymphoma (MCL) patients [87]. A possible explanation could be that SIRT3 deacetylates IDH2, which stimulates the production of NADPH in the mitochondria preventing ROS formation. The expression of IDH2 was significantly downregulated in the early stages but upregulated in advanced stages of colon carcinoma compared to peritumor tissues. The growth of colon cancer cell lines was inhibited by IDH2-siRNA and increased after transfection with IDH2 overexpressing plasmids, indicating that IDH2 might play a role in the development of colon carcinoma [88]. In addition, IDH2 expression was reduced in some cancers, such as renal [61], gastric [65], and hepatocellular carcinomas [63], and IDH2 inhibited metastatic invasion of gastric and hepatocellular carcinoma cells via matrix metalloproteinases.

## 7. Research Progress of Wild-Type IDH3 in Cancers

There are no reported IDH3 mutants in tumor cells, and very few studies have explored the role of wild-type IDH3 in cancer. A recent study concluded that inhibition of oxidative phosphorylation (OXPHOS) preferentially kills quiescent melanoma cells through a mechanism in which *c-Myc* selectively activates transcription of genes encoding *IDH3* and many other OXPHOS enzyme subunits, increasing the occupancy of their promoters in melanoma cells [89].

IDH3α could be considered a biomarker for the diagnosis and target of cancer therapy. IDH3α expression levels positively correlate with poor overall survival in human lung and breast cancer. In cervical and lung adenocarcinoma cell lines, silencing of IDH3α increased α-KG levels, which in turn decreased HIF-1 stability, leading to a marked decrease in microvessel density and tumor growth, suggesting an important role for the IDH3-HIF-1 axis in tumor angiogenesis and growth. Similarly, overexpression of IDH3α significantly accelerated the growth of HeLa/IDH3α xenograft tumors in immunodeficient mice. Further metabolomics revealed no change in glucose-derived α-KG before and after IDH3α knockdown, which the authors interpreted as the existence of an IDH3-dependent and glycolytic-independent metabolic pathway [90]. Interestingly, inhibition of IDH3α resulted in an increase rather than a decrease in α KG expression, which is possibly due to the compensatory production of α-KG by IDH1 and IDH2.

IDH3α is highly expressed in hepatocellular carcinoma (HCC) tissue and is closely associated with tumor size and the clinicopathological stage of HCC. IDH3α promotes the upregulation of the expression of Metastasis Associated 1 (MTA1), an oncogene that stimulates epithelial-mesenchymal transition in HCC, which in turn induces cancer cell migration and invasion [67].

IDH3α is increased in samples from GBM patients compared to normal brain tissue. It promotes GBM progression in a mouse model of in situ glioma in which the loss of function of IDH3α not only reduces TCA activity but also promotes methionine cycle activity, S-adenosylmethionine production, and DNA methylation [68].

In summary, IDH3α has been less studied in tumors, and the specific mechanism of its increased expression in tumors is unknown. Cellular metabolism is lattice-like and coordinated; the overlapping functions of IDH enzymes can lead to compensatory effects that must be taken into account.

## 8. Conclusions (Future Perspectives)

Research on mutant IDH1 and IDH2 has increased in recent decades. Many subtypes of inhibitors have shown promising clinical outcomes, resulting in significant and rapid progress from concept to clinic. However, there are no cancer therapy drugs targeting wild-type IDH1/2/3, as the mechanism of wild-type IDH in tumors is only beginning to be understood. Studies have found that changes in the expression level of wild-type IDH in tumors are closely related to prognosis, metastasis, and invasion of various cancers. Therefore, understanding the mechanisms of wild-type IDH alterations will be of great scientific importance and clinical value, both at the therapeutic level and for prognostic assessment.

There are a few studies on wild-type IDH, which are further limited to metabolic changes. Although metabolic dysregulations and cellular energy changes are among the most important biological features of tumor cells, a more detailed study of the relationship between metabolite changes and epigenetics, especially to metabolites that regulate gene expressions, can provide new strategies to prevent tumor growth. For example, deacetylation by SIRT3 increases IDH2 activity, which could protect tumor cells from oxidative stress. Downregulation of IDH3α decreases the effective level of α-KG by reducing the ratio of α-KG to fumarate and succinate, resulting in PHD2 inhibition and HIF-1α protein stabilization [91]. Knockdown of succinate receptor1 (Sucer1) or blockage of SUCNR1 in vitro and in vivo could reverse the effects of succinate by modulating the PI3K-AKT/HIF-1α pathway. Plasma succinate levels correlate significantly with intestinal ischemia/reperfusion-related lung injury after cardiopulmonary bypass [92]. The expression level of IDH1, IDH2, and IDH3 also differ in various tissues, and whether their altered expression is related to the site of tumorigenesis is also unclear. As many tumors show increased IDH1 and IDH2 expression, common pathways and evidence of commonalities in the different tumors might exist, which can then be studied using multiple tumor lines. Evaluating the mechanisms of IDH alteration across different tumor subtypes could help clarify their biological characteristics to improve treatment. Moreover, IDH is closely related to tumor grading and staging, and this dynamic alteration could be studied in depth to intervene early and predict tumor development.

Furthermore, conventional cell culture experiments performed at 20–21% O_2_ (normoxia) do not mimic physiological conditions; therefore, conventional culture conditions are performed in a hyperoxic environment, generating oxidative stress that resembles high glucose conditions. Therefore, in vitro culture of tumor cells should mimic physiological conditions as much as possible to have more valuable and meaningful information. The three IDH subtypes are always in dynamic equilibrium; if one is altered, the others respond in some surrogate fashion to changes in cellular requirements. Although substantial advances have been made in understanding the molecular pathogenesis and biology of wild-type IDH in tumors, this has not translated into significant improvements in patient prognosis. Our increased understanding of IDH enzymes would help to transform the treatment of patients with oncologic diseases and improve survival.

## Figures and Tables

**Table 1:** The expression of wild-type IDHs in cancers. The expression levels of IDHs in tumors not listed in the table but mentioned in the text have not been clearly studied.

IDH Type	Overexpressed	Downregulated
IDH1	Lung adenocarcinoma [55]	Kidney cancer [56]
	Angioimmunoblastic lymphoma [57]	
	Peripheral T cell lymphoma [57]	
	Diffuse large B cell lymphoma [57]	
	Glioblastoma [58]	
	Pancreatic ductal adenocarcinoma [59]	
IDH2	Lung cancer [60]	Kidney cancer [61]
	Ovarian cancer [62]	Hepatocellular carcinoma [63]
	Breast cancer [64]	Gastric cancer [65]
	Endometroid carcinomas [66]	Glioblastoma [15,16]
		Acute lymphocytic leukemia [13]
IDH3	Hepatocellular carcinoma [67]	
	Glioblastoma [68]	
